# Intermittent Superior Vena Cava Syndrome Secondary to Malignant Pericardial Mesothelioma

**DOI:** 10.7759/cureus.12107

**Published:** 2020-12-16

**Authors:** Sarah A Alwusaibie, Jaffar S Alsayigh, Dunya Alfaraj, Abdullatif M Alomair, Sarah A Alsaeed

**Affiliations:** 1 Emergency Department, King Fahad University Hospital, Al Khobar, SAU; 2 Emergency Department, Imam Abdulrahman Bin Faisal University, King Fahad University Hospital, Al Khobar, SAU; 3 Cardiology/Echocardiography, King Fahad University Hospital, Al Khobar, SAU

**Keywords:** svcs, facial swelling, mesothelioma, sarcomatoid, intermittent

## Abstract

Malignant pericardial sarcomatoid mesothelioma is a massively rare tumor accounting for 0.8% of all cases of mesothelioma. Superior vena cava syndrome (SVCS) occurs due to a partial obstruction or compression to the superior vena cava, which hinders the blood outflow to the upper body. It can be caused by an intrinsic factor such as thrombosis, or by an extrinsic factor such as tumors. Clinical presentation includes edema of the face and upper limbs, plethora, dyspnea, dysphagia, stridor and cough. we are reporting a case of a 56-year-old female, who is a known case of hypertension on angiotensin-converting enzyme inhibitors (ACEIs). Presented to the emergency department with intermittent facial swelling and dyspnea. Imaging and pathology reports confirmed the diagnosis of intermittent SVCS secondary to pericardial sarcomatoid mesothelioma with pericardial effusion. What makes our case unique is that both the etiology and the presenting complaint are rare entities, as most SVCS cases are continuously symptomatic throughout the disease course, and are usually caused by a lung cancer or lymphoma.

## Introduction

Superior vena cava is a large vein that drains blood from upper trunk, head and neck. Superior vena cava syndrome (SVCS) occurs due to an obstruction of the vein, which is characterized by cyanosis, plethora and distention of sub-cutaneous vessels, in addition to an edema of the arms, head and neck. The resulting edema may jeopardize the airways causing dyspnea, stridor and cough. The obstruction can be due to intrinsic etiologies such as a thrombus, or a long-term indwelling catheter. It can also be due to extrinsic pathologies such as tumors, or in rare occasions pericardial effusion. It could be a life-threatening condition as it may compromise cardiac output and results in shock [1]. We are reporting a case of an intermittent SVCS caused by pericardial sarcomatoid mesothelioma with pericardial effusion. Both the presentation and the etiology are rare entities. We reviewed the literature and found two cases similar to ours in respect to their presentation, and two others in regard to the etiology.

## Case presentation

**Figure 1:**
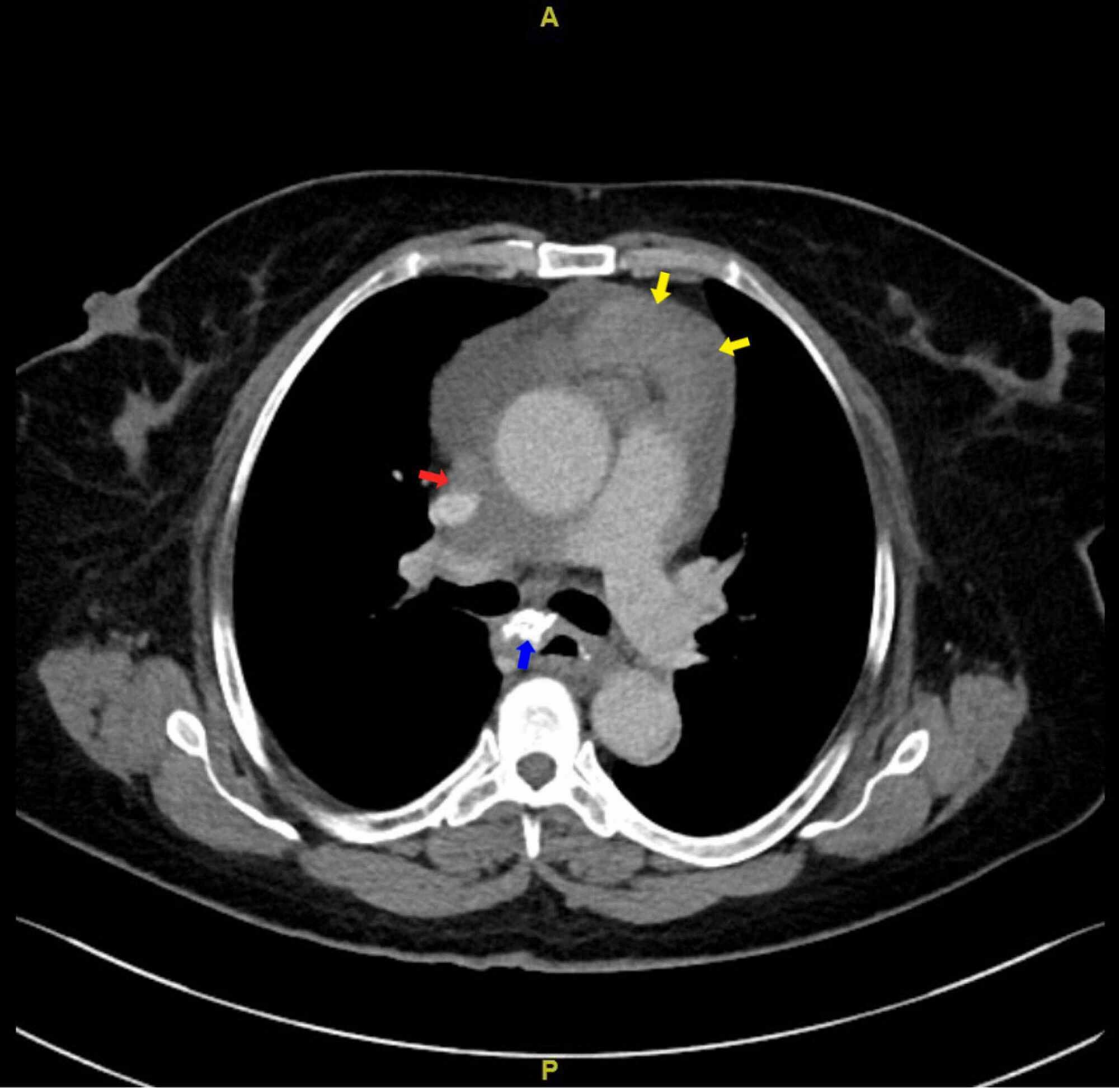
CT chest axial view: red arrow showing soft tissue density encasing SVC causing narrowing; yellow arrows showing node like mass seen in the interior mediastinum; blue arrow pointing at large calcified lymph node. Also, pericardial effusion is noted. CT: computed tomography; SVC: superior vena cava.

**Figure 2:**
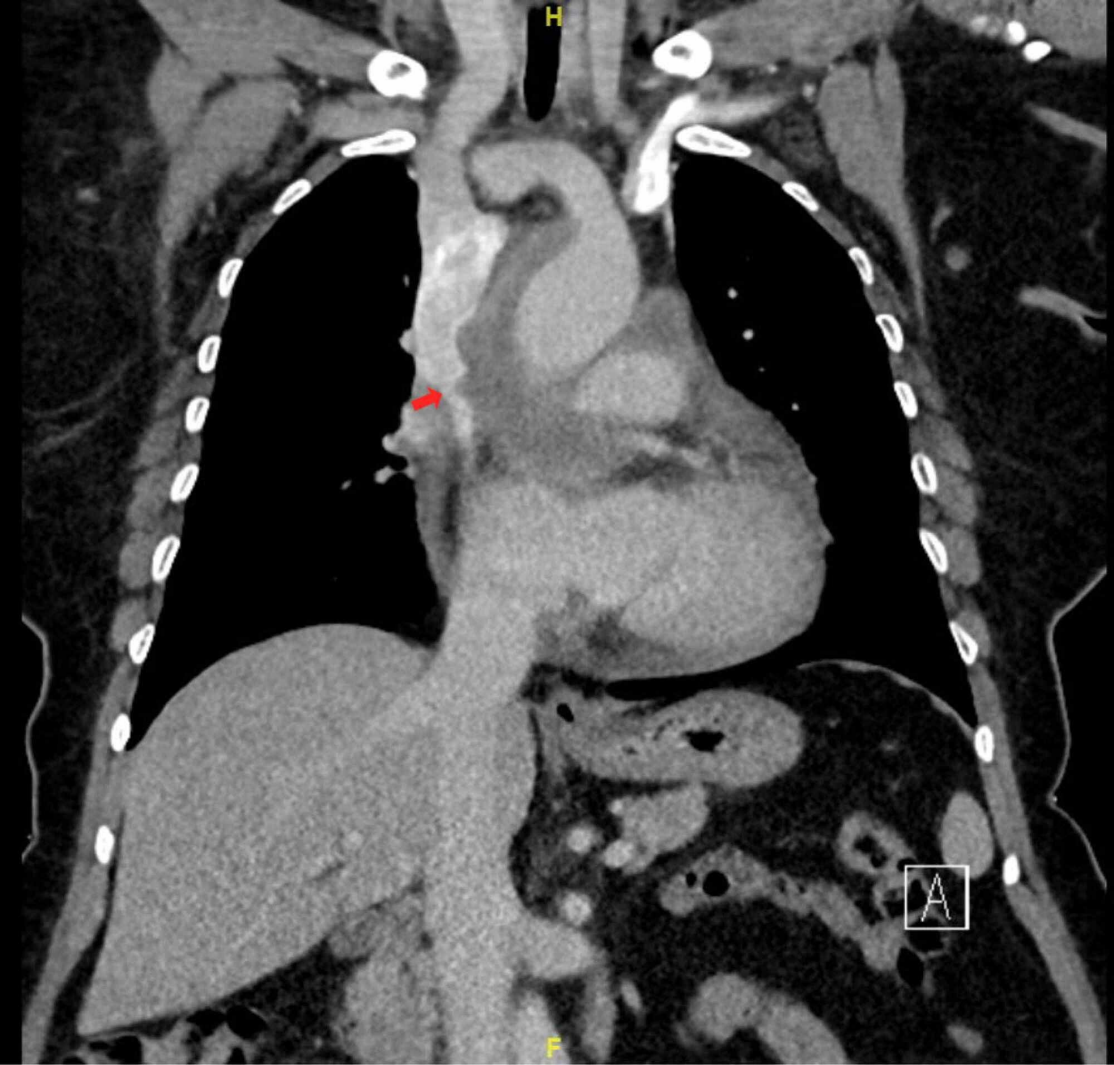
CT chest coronal view: red arrow showing SVC narrowing, pericardial effusion is also noted here. CT: computed tomography; SVC: superior vena cava.

A 56-year-old female, known case of hypertension and dyslipidemia for more than 10 years on Amlodipine and Valsartan, presented to the emergency department of a tertiary hospital with intermittent facial swelling and exertional dyspnea for a one-week duration. In the context of severity, she described her face as a balloon in the morning after sleeping eight hours or more, and by the end of the day only minimal swelling around the eyes remains, not associated with change in color, headache, hoarseness or dysphagia. Patient reported loss of weight around 10 kg in a two-month period. Furthermore, her dyspnea is not associated with chest pain, palpitation, orthopnea, dizziness, syncope, cough or fever. The patient denied any history of trauma, allergies, urinary or gastrointestinal symptoms. She reported multiple emergency visits for the same complain, the patient was investigated, reassured and discharged in each visit. Upon examination, she was conscious, alert, oriented to time, place and person, not in pain but in mild respiratory distress. She wasn’t pale, cyanosed or jaundiced, but there was a mild swelling around the eyes and lips. Her vital signs were as follows: temperature: 37.0 °C, pulse: 101/min, respiratory rate: 24, blood pressure: 146\86 mmHg, Oxygen saturation: 96%, on room air. Chest, abdomen, upper and lower limbs examinations were unremarkable. All her labs were insignificant except for microcytic hypochromic anemia, the labs were as follow: complete blood count (CBC) showed a white blood cells (WBC): 5.3 k/ul (normal 4-11 k/ul), hemoglobin: 8.7 g/dl (normal 12-16 g/dl), MCV: 64.4 fL (normal 80-94 fL), MCH: 19.8 pg (normal 27-32 pg), RDW: 15.9% (normal 11.5%-14.5%), platelets: 340 k/ul (normal 140-450 k/ul), liver function test (LFT): Total Bili: 0.4 mg/dl (normal 0.2- 1.2 mg/dl), Direct Bili: 0.2 mg/dl (normal 0.1- 0.5 mg/dl), albumin: 3.8 g/dl (normal 3.2- 5.2 g/dl). Alkaline phosphatase (ALP) 169 IU/L (normal 40-150 IU/L), all other liver enzymes were within the normal range. Renal function test (RFT): BUN: 5 mg/dl (normal 7-26 mg/dl), creatinine: 0.61 mg/dl (normal 0.6-1.3 mg/dl), all electrolytes were within normal range. Chest X-ray and echocardiogram were suggestive of a moderate pericardial effusion. Computed tomography (CT) scan showed matted low-density soft tissues noted in the prevascular space and surrounding the superior vena cava, which was markedly narrowed. A large calcified lymph node was noted at the sub-carina as well, which rendered a histopathological testing (Figures 1, 2).

Patient was admitted, and on her fifth day of admission she underwent video-assisted thoracoscopic surgery (VATS) for biopsy of the soft matted lesion found on the CT scan. At first, 200cc hemorrhagic pericardial fluid was drained. Then, the surgeon found a mass felt within the pericardium compressing the left atrium. Upon retracting the mass, the left pulmonary artery was injured, the injury had extended to the main pulmonary artery which resulted in massive bleeding. Therefore, the procedure was converted to open thoracotomy. The patient was hypotensive, and developed cardiac arrest, open cardiac massage was performed with suction and compressions. The tear was clamped, bleeding was controlled, and the patient was resuscitated. A superficial wedge biopsy then was taken. Shortly after, the patient re-bled from the same source and developed hypotensive shock, then got a second cardiac arrest. Unfortunately, she was announced dead at the end of the procedure. Moreover, histopathology report was positive for pan CK, CK7, calretinin, focally weakly for D2-40 and CD56. Ki67: high with a percentage of 70%. It is highly suggestive of a sarcomatoid mesothelioma.

## Discussion

Malignant pericardial sarcomatoid mesothelioma is a massively rare tumor accounting for 0.8% of all cases of mesothelioma. Most mesotheliomas histopathological types are either epithelioid or biphasic which usually occur in the pleura [2]. It can also arise from the lining of other structures such as peritoneum, tunica vaginalis of the testis and to a lesser extent the pericardium. The diagnosis is made by a biopsy, but more commonly from an autopsy [3].

SVCS occurs due to a partial obstruction or compression to the superior vena cava, which hinders the blood outflow to the upper body. It is characterized by cyanosis, plethora, distention of sub-cutaneous vessels, and edema of the arms, head and neck. The resulting edema may jeopardize the airways causing dyspnea, stridor and cough. The obstruction can be due to intrinsic etiologies such as a thrombus, or a long-term indwelling catheter. while extrinsic pathologies can be caused by tumors, or very rarely a pericardial effusion. Small cell lung cancer and non-Hodgkin lymphoma are the most common culprits. It could be a life-threatening condition as it may compromise cardiac output and results in shock [3,4]. Intermittent facial swelling is an uncommon presenting complain, and it can be caused by a variety of etiologies. The most common of which is angioedema. Nonetheless, more serious etiologies should be ruled out such as SVCS [5].

A PubMed (US National Library of Medicine, Bethesda, MD, USA) search using medical subject headings (MeSH) “intermittent facial swelling”, “superior vena cava syndrome” and “pericardial sarcomatoid mesothelioma” resulted in four cases that have similarities to our case. 

The first case was reported in 2014 for an 84-year-old man who is a known case of atrial fibrillation, coronary bypass graft, chronic bronchitis, dementia and ex-smoker (60 packs per year), and had worked as a miner. He presented with intermittent face and right arm swelling for eight weeks, that is worse in the morning, and improved as the day went on. He also reported shortness of breath and right wrist pain. At first, a diagnosis of angioedema was made. However, after further investigations, a final diagnosis with SVCS secondary to metastatic bronchogenic carcinoma was established [5].

The second case was in 1992 for a 48-year-old male who is a known case of Hodgkin’s lymphoma in the neck which was metastasized to the chest, abdomen and pelvic lymph nodes. Hickman catheter was inserted for him via the left subclavian vein to gain a vascular access for administration of chemotherapy. The catheter’s tip was extending to the SVC vein. Several months later, he presented with intermittent tightness of the neck and distension of the veins on the forehead specially when bending down. Further investigations revealed an intermittent SVCS caused by a thrombus at the end of the catheter, acting as a ball valve [6].

The third case was in 2009. It is regarding a 60-year-old woman presented with a repeated dry cough, exertional dyspnea and shortness of breath due to a large pericardial effusion and bilateral pleural effusion. There was no exposure to asbestos, tuberculosis or smoke. Shortly after her presentation, the patient developed a superior vena cava compression along with pericardial constriction. Partial pericardiectomy revealed a large tumor which was then attributed to pericardial mesothelioma [7].

The last case was published in 2015 for a 70-year-old woman, status post right lower lobectomy and chemotherapy for adenocarcinoma six years prior to presentation. Presented with a history of facial swelling for three weeks. CT scan revealed a loculated pericardial effusion compressing the SVC. Pericardiocentesis rapidly relieved her symptoms and fluid analysis later on confirmed a malignant effusion [8].

In comparison to the above cases, we found several similarities to our case. In settings of presentation, the first two cases were both presented with symptoms of intermittent SVCS due to different etiologies. The third case is similar to ours in that they’re both caused by the same tumor that is mesothelioma. The last case was documented to explain why we thought of pericardial effusion as an attributive factor to the patient’s clinical manifestations. 

In our patient, we first thought of angioedema as the number one differential diagnosis as the patient was using ACEIs. Nevertheless, this is a diagnosis of exclusion thus other diagnoses must be ruled out first including SVCS. Based on the radiological and histopathological reports, we concluded that the most likely cause of her symptoms is pericardial mesothelioma with malignant pericardial effusion.

In terms of management, SVCS is an etiology based management. Therefore, malignant pericardial mesothelioma will be addressed. The disease follows an extremely poor prognosis with a median survival rate of six months. Standardized treatment guidelines for it are yet to be established. However, current practice implies that patients with an early stage of the disease or a loculated one might benefit from surgical therapy, yet most patients are diagnosed at an advanced stage due to the absence of symptoms early on. Systemic chemotherapy is indicated for those with an advance or unresectable disease. Having said that, the sarcomatoid histopathological subtype of mesothelioma has shown a very poor response to chemotherapy. additionally, pericardiocentesis is commonly done to alleviate heart failure onset. On those grounds, Further improvement in the therapeutic modalities used for malignant pericardial mesothelioma is necessary [3].

## Conclusions

In summary, SVCS can present with intermittent symptoms on rare occasions. It could represent a fatal diagnosis hence, it shouldn’t be taken lightly and must be ruled out first before considering other diagnoses. Furthermore, mesothelioma can be a rare cause for this unusual presentation.
